# The association between human papillomavirus infection and lung cancer: a system review and meta-analysis

**DOI:** 10.18632/oncotarget.21682

**Published:** 2017-10-09

**Authors:** Wei-Min Xiong, Qiu-Ping Xu, Xu Li, Ren-Dong Xiao, Lin Cai, Fei He

**Affiliations:** ^1^ Department of Epidemiology and Health Statistics, School of Public Health, Fujian Medical University, Fuzhou, 350108, China; ^2^ Fujian Provincial Key Laboratory of Tumor Microbiology, Fujian Medical University, Fuzhou, 350108, China; ^3^ Key Laboratory of Ministry of Education for Gastrointestinal Cancer, Fujian Medical University, Fuzhou, 350108, China; ^4^ Department of Thoracic Surgery, The First Affiliated Hospital of Fujian Medical University, Fuzhou, 350001, China

**Keywords:** lung neoplasms, human papillomavirus, meta-analysis, case-control study, cohort study

## Abstract

To estimate the global attributable fraction of human papillomavirus (HPV) in lung cancer, we provided updated information through a system review and meta-analysis. We did a literature search on PubMed, Ovid and Web of Science to identify case-control studies and cohort studies that detected HPV in lung carcinomas. We included studies that tested 30 or more cases and were published before Feb 28, 2017. We collected information about gender, smoking status, HPV detection methods, HPV types, materials and clinical features. If it was not possible to abstract the required information directly from the papers, we contacted the authors. A meta-analysis was performed to calculate the pooled effect sizes (*OR/RR*) with 95% confidence intervals (*CI*) including subgroup analysis and meta-regression to explore sources of heterogeneity, by Stata 13.0 software. 36 case-control studies, contributing data for 6,980 cases of lung cancer and 7,474 controls from 17 countries and one cohort study with 24,162 exposed and 1,026,986 unexposed from China were included. HPV infection was associated with cancer of lung, pooled *OR* was 3.64 (95% *CI*: 2.60–5.08), calculated with the random-effects model. Pooled *OR* for allogeneic case-control studies, self-matched case-control studies and nested case-control studies were 6.71 (95% *CI*: 4.07–11.07), 2.59 (95% *CI*: 1.43–4.69) and 0.92 (95% *CI*: 0.63–1.36), respectively. Pooled OR for HPV 16 and HPV 18 infection, were 3.14 (95% *CI*: 2.07–4.76) and 2.25 (95% *CI*: 1.49–3.40), respectively. We also found that HPV infection may be associated with squamous cell carcinoma, adenocarcinoma and small cell carcinoma. There is evidence that HPV infection, especially HPV 16 and HPV 18 infection, significantly increase the risk of lung cancer. Future research needs to focus attention toward whether an HPV vaccine can effectively reduce the incidence of lung cancer.

## INTRODUCTION

By 2012, cancer holds the first place of morbidity and mortality in the worldwide, with 14 million new cases and 8 million deaths, which means the age-standardized incidence and mortality were 182 and 102 per 100 000, respectively [1]. According to the International Cancer Research Center, the latest data shows that lung cancer still accounts first for the world's cancer incidence and death in 2012 [2], but also the first cause of incidence and death of malignant tumors in China [3]. Particularly, lung cancer for men had the highest incidence (34.2 per 100 000) and mortality (30.0 per 100 000) and for women had the fourth highest incidence (13.6 per 100 000) and the second highest mortality (11.1 per 100 000) [1].

Besides dietary factors and tobacco smoke, infectious diseases represent the third leading cause of cancer in the entire world and the proportion of cancers associated with pathogenic microorganism was estimated to be 16.1% [4]. As well known, infection with HPVs is the risk factor of almost all cervical cancer [5], most of the anus - genital cancer and more than a quarter of oropharyngeal cancer [6]. In fact, it appears that the chronic infections with HPV should be responsible for approximately the 5% of all human cancers [7]. A dozen of HPV types, including types 16, 18, 31, 33, 35, 39, 45, 51, 52, 56, 58, and 59, have been allocated by International Agency for Research on Cancer in Group 1, as their carcinogenicity to humans has been sufficiently demonstrated. Other HPV types are categorized either in Group 2A (probably carcinogenic), Group 2B (possibly carcinogenic) or Group 3 (inadequate evidence of carcinogenicity to humans).

HPV belongs to the papillomaviridae, a large family of epitheliotropic DNA viruses. HPV gene expression and the viral life cycle are tightly controlled by epithelial cell differentiation. It is assumed that scratching of the epithelial tissue allows the virus to infect undifferentiated cells in the basal layers of stratified squamous epithelium [8]. According to the character of HPV, which has a high degree of affinity to the squamous epithelium and the feature of bronchus and lung, whose main tissue type was epithelial tissue, so it is postulated that HPV is probably related with lung neoplasms.

It has been 38 years since Syrjänen who first suggested that HPV could possibly be involved in bronchial squamous cell carcinoma [9]. But in the follow-up studies, the results were not consistent. In worldwide, HPV infection rate was 0–78.3% in lung cancer [10]. There is an enormous difference of infectious rate of HPV in different regions. At the meantime, differences in sensitivity and specificity of HPV genotyping methods and diagnostic criteria, in addition to the limited spectrum of HPV types analyzed may all contribute to the inconsistent result. The current epidemiological study of the relationship between HPV infection and lung cancer is controversial, and the associated meta-analysis is mainly limited to a single study type or the rate of HPV infection in patients with lung cancer. Herein, this systematic review and formal meta-analysis aimed at conducting a quantitative evaluation for the strength of the association between the lung neoplasms and HPV infection, by searching published analytic epidemiologic studies at home and abroad.

## RESULTS

### Eligible studies characteristics

**Figure 1:**
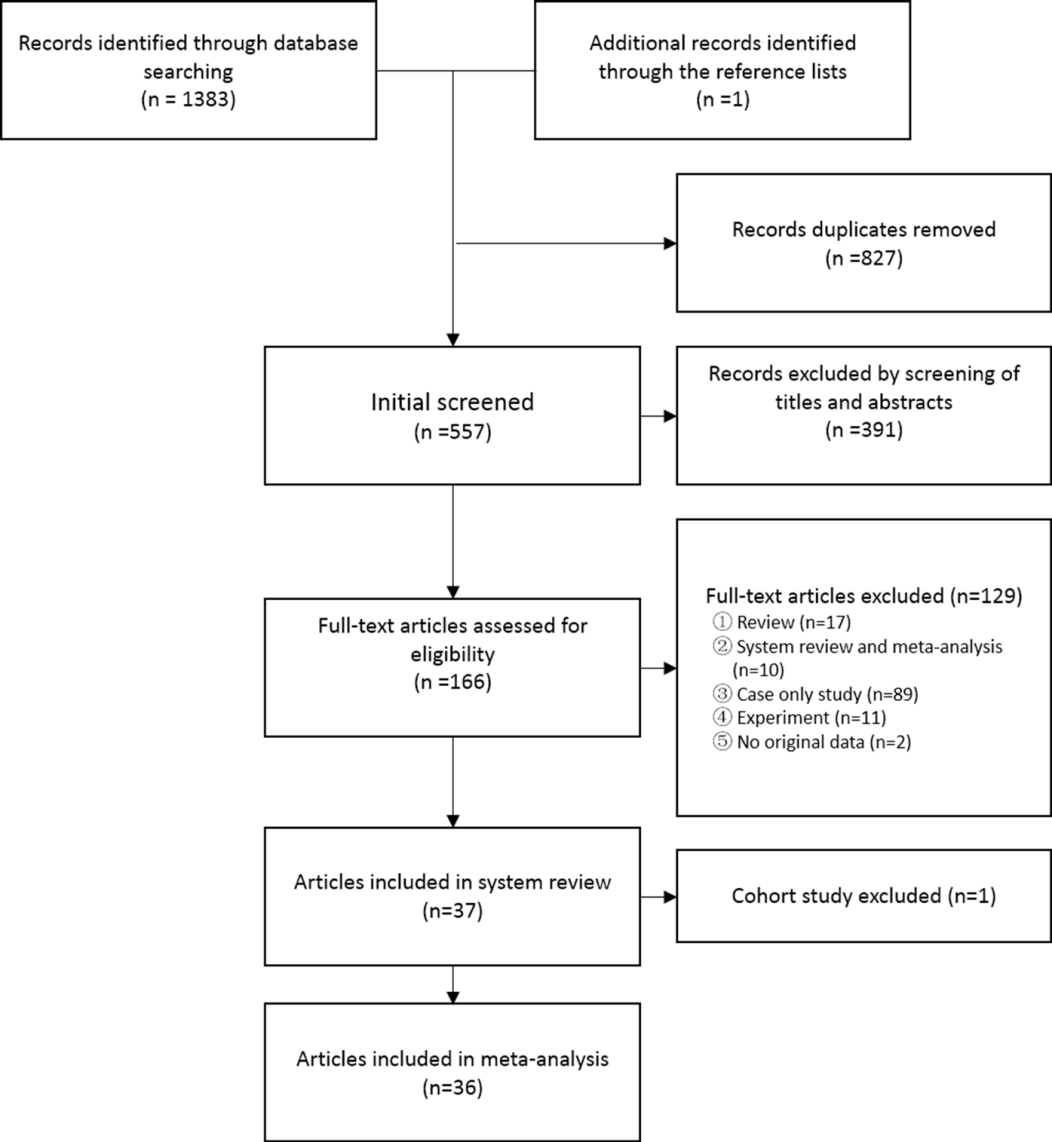
Flow diagram of systematic literature search

According to the established search strategy and inclusion criteria, after screening and detailed examination, a total of 37 eligible articles were selected (Figure 1). 36 of them were case-control studies (24 allogeneic case-control studies [11–34], 8 self-matched case-control studies [35–42], 3 nested case-control studies [43–45] and 1 nested and allogeneic case-control study [46]), including 6,980 cases of lung cancer and 7,474 controls. One population-based cohort study from China was also included. In that study, the incidence of lung cancer in 24,162 HPV-infected patients was compared to 1,026,986 uninfected individuals. After adjusting for age, sex, income, residence and concomitant diseases, there was a significant increase in the risk of lung cancer among women who were exposed to HPV infection (*RR* = 1.263, 95% *CI*: 1.015–1.571, *P* = 0.0367), while no significant association was found among men (*RR* = 1.169, 95% *CI*: 0.984–1.390, *P* = 0.0754). However, this cohort-study lacked information on individual lifestyle, smoking, diagnostic criteria for HPV infection, and detailed HPV types.

36 case-control studies were conducted further meta-analysis. The majority of cases in the pooled dataset were from Europe (61.2%, 4,274 cases) and Asia (33.2%, 2,315 cases). North American studies represented 4.1% of all cases in the pooled dataset (*n* = 287) and Oceania studies represented 1.5% of all cases in the pooled dataset (*n* = 104). The size of studies in the pooled dataset varied from 30 to 1,634 cases. The full list of the included case-control publications was shown in Table 1.

**Table 1:** The basic information of the selected literature

Author_year	Country	Method	HPV types	Sample type	Case (n/N)	Control (n/N)
Béjui-Thivolet_1990	French	ISH	6, 11, 16, 18	tissue	6/33	0/10
Li_1995	China	PCR, DB	16, 18	tissue	16/50	0/22
Fong_1995	Australia	PCR	6, 11, 16, 18, 31, 33, 52b, 58	tissue	2/104	0/104
Yang_1998	China	PCR	6/11, 16, 31/33	tissue	13/50	3/30
Niyaz_2000	China	PCR	16, 18	tissue	44/110	1/40
Cheng_2001	China	PCR, ISH	16, 18	tissue	77/141	16/60
Chiou_2003	China	PCR	16, 18	blood	71/149	22/174
Cheng_2004	China	PCR, ISH	6, 11	tissue	40/141	1/60
Jain_2005	India	PCR	16, 18	tissue(case) blood(control)	2/40	0/40
Ciotti_2006	Italy	PCR, sequencing	16, 18, 31	tissue	8/38	0/38
Fei_2006	China	ISH	16, 18	tissue	23/73	2/34
Giuliani_2007	Italy	PCR, reverse blot hybridization, sequencing	-	tissue	10/78	0/78
Nadji_2007	Iran	PCR, sequencing	-	tissue	33/129	8/89
Buyru_2008	Turkey	PCR, SB	16, 18	blood	1/65	0/87
Wang_2008	China	PCR, ISH, IHC	16, 18	tissue	138/313	4/96
Yu_2009	China	PCR	25 types	tissue	43/109	16/71
Xu_2009	China	ISH	16/18	tissue	32/44	0/15
Krikelis_2010	Greece	PCR	16	tissue, BW	36/58	11/16
Wang_2010	China	PCR	16, 18	tissue	18/45	0/16
Joh_2010	USA	PCR, sequencing	-	tissue	5/30	0/21
Carpagnano_2011	Italy	PCR, sequencing, INFINITI HPV-QUAD assay	16, 18, 30, 31, 33, 45, 35/68, 39/56, 58/52, 59/51, 6/11	tissue, BW, EBC	12/89	0/68
Galvan_2012	Italy, UK	PCR,DB	35 types	tissue	0/100	0/100
Gatta_2012	Italy	PCR	16, 18, 33, 35, 52, 58	tissue	2/50	1/23
Yu_2013	China	PCR, reverse blot hybridization, SB	25 types	tissue	75/170	21/91
Anantharaman_2014	7 European countries	BMSM	6, 11, 16, 18, 31	blood	791/1634	991/2729
Sagerup_2014	Norway	PCR	15 types	tissue	13/334	0/13
Sarchianaki_2014	Greece	PCR, genotyping	37 types	tissue	19/100	0/16
Yu_2015	China	PCR	L1, 16, 18	tissue	100/180	8/110
Fan_2016	China	ICC	16	PE	42/95	1/55
Gupta_2016	India	PCR	16, 18, 31, 33, 45	FNAC, tissue	5/73	0/75
Lu_2016	China	PCR	16, 18	tissue	33/72	2/54
Robinson_2016	USA	microarray, oncovirus panel, genotyping PCR	28 types	tissue	15/57	1/10
Xiong_2016	China	PCR, reverse blot hybridization	21 types	tissue	7/83	6/83
Simen_2010	Finland	ELISA	16, 18	serum	67/311	220/930
Anantharaman_2014	10 European countries	BMSM	6, 11, 16, 18, 31	blood	604/1449	601/1599
Colombara_2015	USA	LBMA	6, 11, 16, 18, 31, 33, 52, 58	serum	4/200	15/200
Colombara_2016	China	LBMA	6, 11, 16, 18, 31, 33, 52, 58	serum	8/183	8/217

Abbreviations: Author_year, name of first author_year of publication; Case(n/N), Case (number of HPV positive cases/number of cases); Control (n/N), Control (number of HPV positive controls/number of controls); ISH, *In situ* hybridization; PCR, Polymerase chain reaction; DB, Dot blot; SB, Southern blot; IHC, Immunohistochemistry; BMSM, Bead-based multiplex serology method; ICC, Immunocytochemistry; ELISA, Enzyme-linked immunosorbent assay; LBMA, Multiplex liquid bead microarray antibody assay; BW, bronchial washing; EBC, exhaled breath condensate; PE, pleural effusion; FNAC, fine-needle aspiration cytology.

### HPV infection and lung neoplasms pooled risk

**Figure 2:**
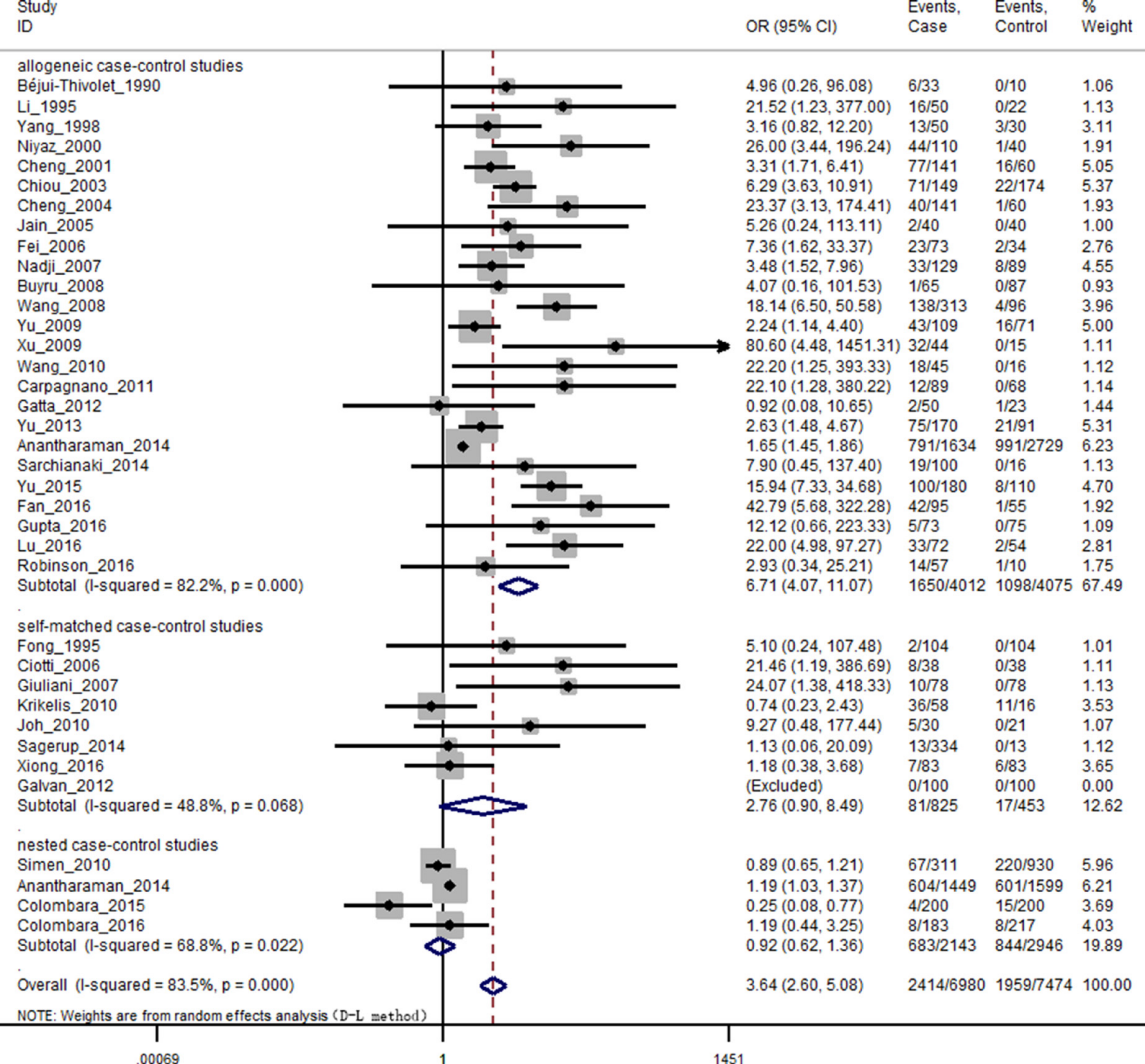
Forest plot of random effects model stratified by study design Individual study *OR* (squares) and *OR*_sub_ (diamonds) values are plotted with 95% confidence intervals (LCL, lower confidence limit; UCL, upper confidence limit) for each study design. Symbol sizes reflect relative weight of the studies.

The heterogeneity test results of case group and control group were χ^2^ = 212.51, *P* < 0.001, *I*^2^ = 83.5%. Due to *P* < 0.1, *I*^2^ > 50%, we used a random effects model meta-analysis and pooled *OR* and 95% *CI*: 3.64 (2.60–5.08), *P* < 0.001, estimate of between-study variance Tau^2^ = 0.47 (Figure 2).

The results of subgroup analysis were shown as follows: By the study design, using random effects model for allogeneic case-control studies *P* < 0.001, *I*^2^ = 82.2%, pooled *OR* as 6.71 and 95% *CI*: 4.07–11.07; using fixed effects model for self-matched case-control studies *P* = 0.07, *I*^2^ = 48.8%, pooled *OR* as 2.59 and 95% *CI*: 1.43–4.69; using random effects model for nested case-control studies *P* = 0.02, *I*^2^ = 68.8%, pooled *OR* as 0.92 and 95% *CI*: 0.63–1.36 (Figure 2).

In different regions: HPV infection in lung cancer patients were significantly higher compared to controls only among the Asian subjects (*OR* = 6.24, 95% *CI*: 3.88–10.04, *P* < 0.001). Significantly higher HPV infection rates were found in lung cancer patients, compared to controls, in both male (*OR* = 3.31, 95% *CI*: 2.02–5.42, *P* < 0.001) and female (*OR* = 3.29, 95% *CI*: 1.01–10.74, *P* = 0.049). And a significant difference in pooled *OR* between male and female was not found in HPV infection and lung cancer (*P* > 0.05). However, significantly higher HPV infection rates were found in lung cancer patients, compared to controls, in non-smokers (*OR* = 6.51, 95% *CI*: 3.78–11.21, *P* < 0.001), but not in smoker subgroup (*P* > 0.05). Stratified by HPV detection method, significantly higher HPV infection rates were found in polymerase chain reaction (PCR), *in situ* hybridization (ISH), southern blot/dot blot (SB/DB), sequencing and bead-based multiplex serology method (BMSM), but not in multiplex liquid bead microarray antibody assay (LBMA). Stratified by HPV type, significantly higher HPV infection rates were found in lung cancer, compared to controls, in both HPV 16 (*OR* = 3.14, 95% *CI*: 2.07–4.76, *P* < 0.001), HPV 18 (*OR* = 2.25, 95% *CI*: 1.49–3.40, *P* < 0.001) and HPV 11 (*OR* = 1.30, 95% *CI*: 1.12–1.52, *P* = 0.001), but not in HPV 6 and HPV 31 (*P* > 0.05). There was a significant difference in pooled *OR* between tissue and blood (*P* < 0.05). Using tissues as detection materials, we found that HPV infection was a risk factor of lung cancer. While using blood, a significant association was not found between HPV infection and lung cancer. We also pooled HPV DNA positive rates in lung cancer patients and controls by sample type (Supplementary Figure 1). There is no significant difference in pooled *OR* between blood (*OR* = 6.43, 95% *CI*: 3.74–11.05, *P* < 0.001) and tissue (*OR* = 6.29, 95% *CI*: 4.01–9.88, *P* < 0.001). Stratified by histological type, significantly higher HPV infection rates were found in lung cancer, compared to controls, in both adenocarcinoma (*OR* = 5.39, 95% *CI*: 2.89–10.06, *P* < 0.001), squamous cell carcinoma (*OR* = 5.66, 95% *CI*: 4.38–7.33, *P* < 0.001) and small cell carcinoma (*OR* = 6.74, 95% *CI*: 3.41–13.35, *P* < 0.001), but not in adenosquamous carcinoma and large cell carcinoma (*P* > 0.05). Regardless of the clinical stage and differentiated grade, HPV infection was a risk factor of lung cancer. Subgroup analysis results of continent, gender, smoking, detection method, HPV type, material, histological type, clinical stage and differentiated grade for the relationships between HPV infection and lung cancer were shown in Table 2.

**Table 2:** Subgroup analysis for the relationships between HPV infection and lung cancer

subgroup	No. of studies	Case (n/N)	Control (n/N)	*I*^2^,%	model	OR (95%CI)	*P*
Continent							
Europe	12	1568/4274	1824/5620	70.7	random	1.37 (0.99–1.90)	0.058
Asia	21	821/2315	119/1519	69.3	random	6.24 (3.88–10.04)	< 0.001
America	3	23/287	16/231	74.7	random	1.44 (0.14–14.31)	0.757
Gender							
male	9	108/675	19/350	0.0	fixed	3.31 (2.02–5.42)	< 0.001
female	10	143/711	225/1104	68.8	random	3.29 (1.01–10.74)	0.049
smoking							
non-smoker	9	103/256	17/203	7.7	fixed	6.51 (3.78–11.21)	< 0.001
smoker	11	117/1048	38/533	57.1	random	1.97 (0.86–4.52)	0.108
detection method							
PCR	28	837/2958	121/1685	59.6	random	5.30 (3.44–8.17)	< 0.001
ISH	3	61/150	2/59	17.9	fixed	12.40 (3.86–39.83)	< 0.001
SB/DB	6	109/546	27/461	41.9	fixed	3.12 (1.95–4.98)	< 0.001
sequencing	4	60/326	8/256	12.6	fixed	5.94 (2.91–12.15)	< 0.001
BMSM	2	1395/3083	1592/4328	91.1	random	1.40 (1.02–1.93)	0.039
LBMA	2	12/383	23/417	76.0	random	0.56 (0.12–2.59)	0.458
HPV type							
16	27	1030/5908	799/6915	80.8	random	3.14 (2.07–4.76)	< 0.001
18	26	732/5828	687/6937	73.7	random	2.25 (1.49–3.40)	< 0.001
6	17	938/4929	964/5549	70.2	random	1.14 (0.81–1.60)	0.461
11	13	357/4526	348/5226	0.0	fixed	1.30 (1.12–1.52)	0.001
31	14	209/4541	296/5347	12.4	fixed	0.96 (0.80–1.16)	0.680
33	6	19/972	23/604	0.0	fixed	0.45 (0.22–0.91)	0.025
material							
frozen tissue	12	310/1373	38/666	50.4	random	5.68 (2.60–12.42)	< 0.001
FFPE tissue	15	479/1325	63/727	64.9	random	6.89 (3.73–12.72)	< 0.001
fresh tissue	2	23/118	0/91	0.0	fixed	17.05 (2.22–131.01)	0.006
blood	7	1546/3991	1857/5936	89.6	random	1.41 (0.95–2.10)	0.088
histological type							
AC	23	277/1191	96/1451	68.8	random	5.39 (2.89–10.06)	< 0.001
SCC	25	348/1156	100/1503	42.7	fixed	5.66 (4.38–7.33)	< 0.001
SmCC	8	24/101	22/587	26.5	fixed	6.74 (3.41–13.35)	< 0.001
ASC	3	1/21	6/203	1.3	fixed	3.04 (0.48–19.47)	0.240
LCC	7	1/18	14/505	0.0	fixed	3.68 (0.53–25.31)	0.186
clinical stage							
I-II	12	226/984	84/874	48.0	fixed	3.53 (2.58–4.84)	< 0.001
III-IV	11	167/536	84/836	26.3	fixed	4.97 (3.60–6.86)	< 0.001
differentiated grade							
well	5	18/95	10/272	0.0	fixed	4.66 (1.93–11.24)	0.001
moderate & low	4	134/369	10/262	0.0	fixed	15.46 (7.90–30.27)	< 0.001

Abbreviations: Case(n/N), Case (number of HPV positive cases/number of cases); Control (n/N), Control (number of HPV positive controls/number of controls); PCR, Polymerase chain reaction; ISH, *In situ* hybridization; SB, Southern blot; DB, Dot blot; BMSM, Bead-based multiplex serology method; LBMA, Multiplex liquid bead microarray antibody assay; FFPE tissue, Formalin fixed and paraffin embedded tissue; AC, Adenocarcinoma; SCC, Squamous cell carcinoma; SmCC, Small cell carcinoma; ASC, Adenosquamous carcinoma; LCC, Large cell carcinoma. Fixed model used Mantel-Haenszel method, random model used DerSimonian-Laird method.

### Meta regression

In single covariate meta-regression, study design, continent, HPV detection method, material, histological type were significant study-level covariates. However, in multi-covariates meta-regression, all the covariates were not significant study-level covariates (data not shown).

### Sensitivity analysis and publication bias

**Figure 3:**
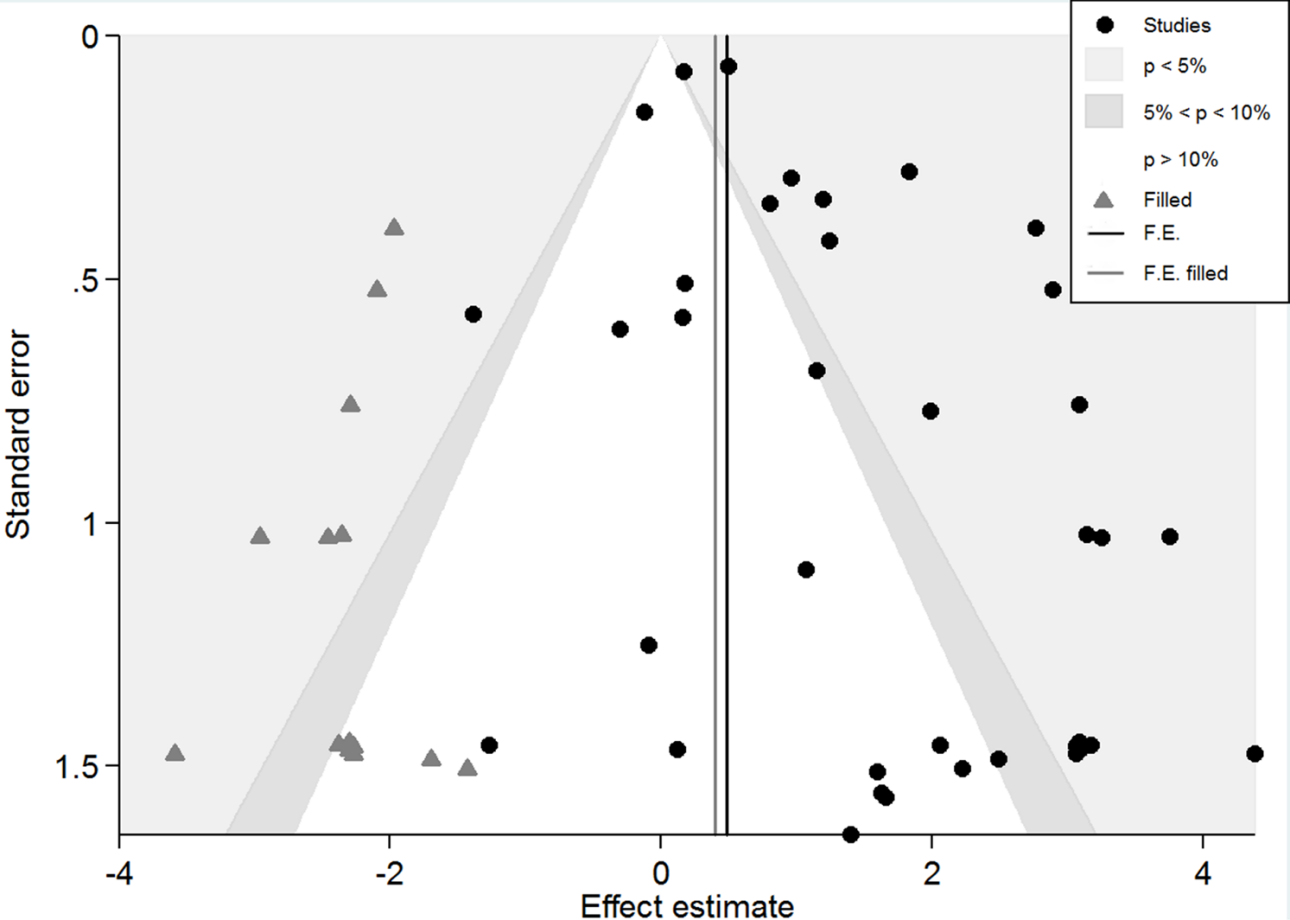
Contour-enhanced meta-analysis funnel plots The vertical black line shows the pooled log odds ratio on the original meta-analysis, while the vertical grey line shows the pooled estimate including the filled studies.

Sensitivity analysis using fixed effects model revealed that there was no significant difference between the studies, and that the effect of a single study removed on the combined results was not significantly changed. When the pooled effect size was compared between fixed effects model (pooled *OR* 95% *CI*: 1.82 (1.69–1.97), *P* < 0.001) and random effects model (pooled *OR* 95% *CI*: 3.64 (2.60–5.08), *P* < 0.001), we found the results of the two models were different, indicating that the small sample studies had an effect on combined effect. Therefore, after we excluded the studies with sample size of case group or control group less than 100, the pooled effect size of fixed effects model (pooled *OR* 95% *CI*: 1.49 (1.37–1.63), *P* < 0.001) was similar with random effects model (pooled *OR* 95% *CI*: 1.87 (1.19–2.95), *P* = 0.007). We used iterative methods to estimate the number of missing studies, and 15 studies were trimmed and filled with recalculating of pooled *OR* and 95% *CI* (Figure 3). The recalculating pooled effect size of fixed effects model (pooled *OR* 95% *CI*: 1.49 (1.37–1.62), *P* < 0.001) and that of random effects model (pooled *OR* 95% *CI*: 1.71 (1.22–2.39), *P* = 0.002) did not occur significantly reversed which meant the results of the meta-analysis were robust. It was less likely that the results would change with new studies reported in the future.

Although some asymmetry occurred in the contour-enhanced funnel plots (Figure 3), Beggs’ rank correlation test suggested no significant publication bias existed (*P*_continuity corrected_ = 0.796). However, the result of Harbord's modified linear regression test (*P* = 0.001) and Peters’ regression test (*P* = 0.011) were significant. Furthermore, the limited number of studies (*n* = 36) indicated a potential publication bias. In Figure 3, trim and fill estimated that 15 studies were missing, all of which indicated those infected HPV were at a reduced risk of lung cancer and eight of which were in the region of *P* > 0.10. Hence, it was plausible that publication bias was a part of the cause of the observed asymmetry in this funnel plot. However, seven of 15 studies were in the region of *P* < 0.05, then confounding factors that cause systematic differences in the results of large and small studies was a likely cause of the funnel asymmetry. Nfs_0.05_ = 2326, which was much larger than the number of eligible studies. Even if there was a publication bias, the results were still relatively stable.

## DISCUSSION

**Figure 4:**
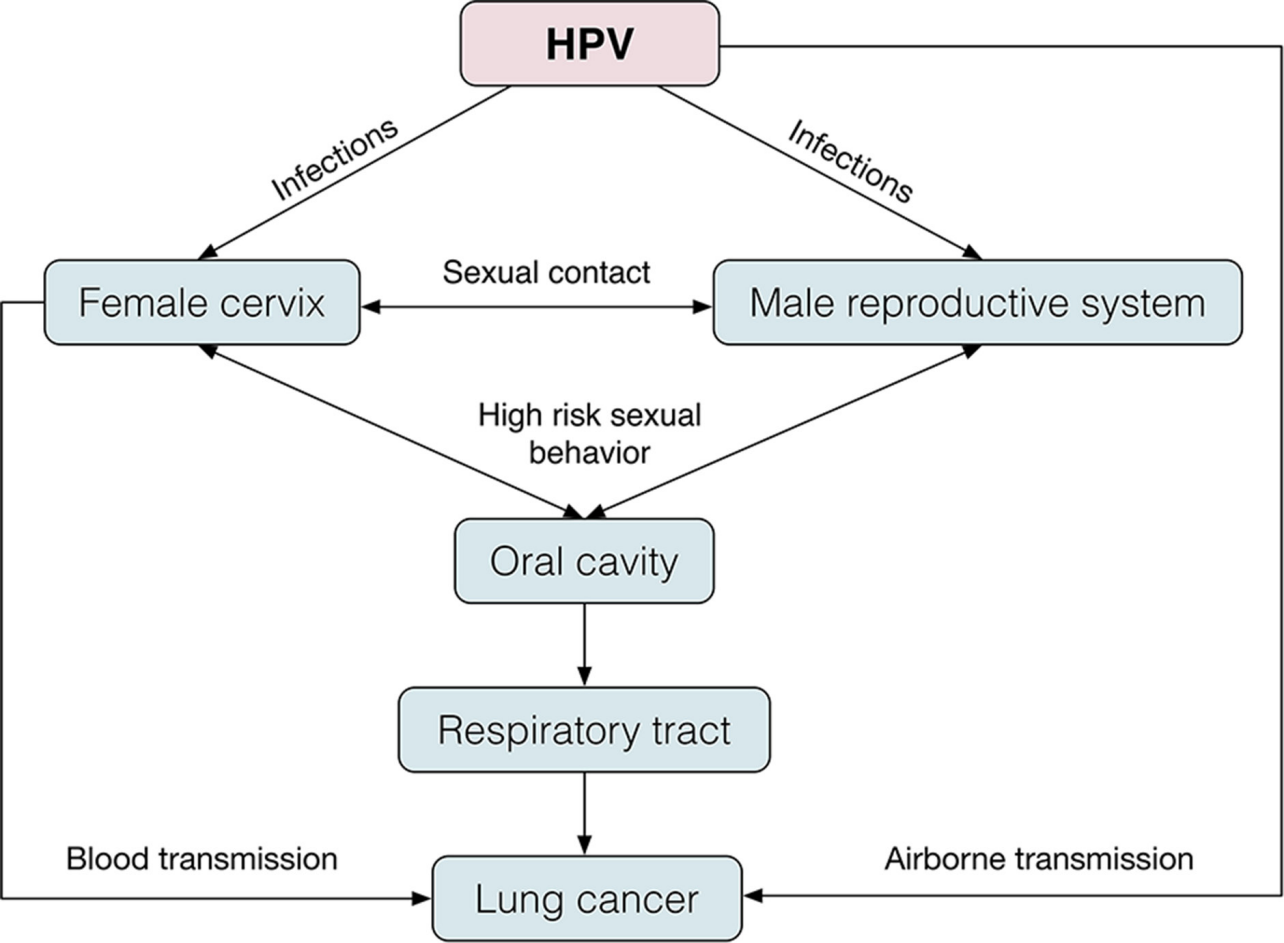
The pathogenesis of HPV infection in thoracic visceral lungs (1) Transmission through the cervical lesion to the lung, (2) high-risk sexual behavior from the infected reproductive system to the mouth and then through the throat into the lungs, (3) through the air to the respiratory system and the lungs.

HPV is a non-coated double-stranded epitheliotropic DNA virus [47]. There are more than 150 types of HPV transmitted through the skin and / or sexual contact [48]. There are three hypotheses regarding the pathogenesis of HPV infection in thoracic visceral lungs (Figure 4): (1) transmission through the cervical lesion to the lung, (2) high-risk sexual behavior from the infected reproductive system to the mouth and then through the throat into the lungs, (3) through the air to the respiratory system and the lungs. Iwamasa et al. reports that approximately 80% of HPV-infected women with lung cancer have cervical intraepithelial neoplasia [49]. The same HPV 16/18 DNA sequence can be detected in cervical smears, peripheral blood lymphocytes, and lung cancer [19], suggesting that HPV is likely to be transported from the cervix to the lung tissue. The lung is rich with endothelium, which can capture the virus and lead to lung cancer [50]. HPV can also be transmitted mouth to mouth or mouth to genitalia. A survey study of 222 men and their female partners finds that the male oral HPV infection rate is 7.2%, and the majority of the female partners of HPV-infected males have either oral or genital HPV infection [51]. Thus, HPV in oral cavity foci may transmit through the throat into the lungs. Carpagnano et al. [26] are the first to report the presence of HPV DNA in exhaled breath condensate samples obtained from patients with lung cancer, suggesting that HPV reaches the lungs through respiratory gas flow. Thus, HPV infection may be transmitted through inhalation.

**Figure 5:**
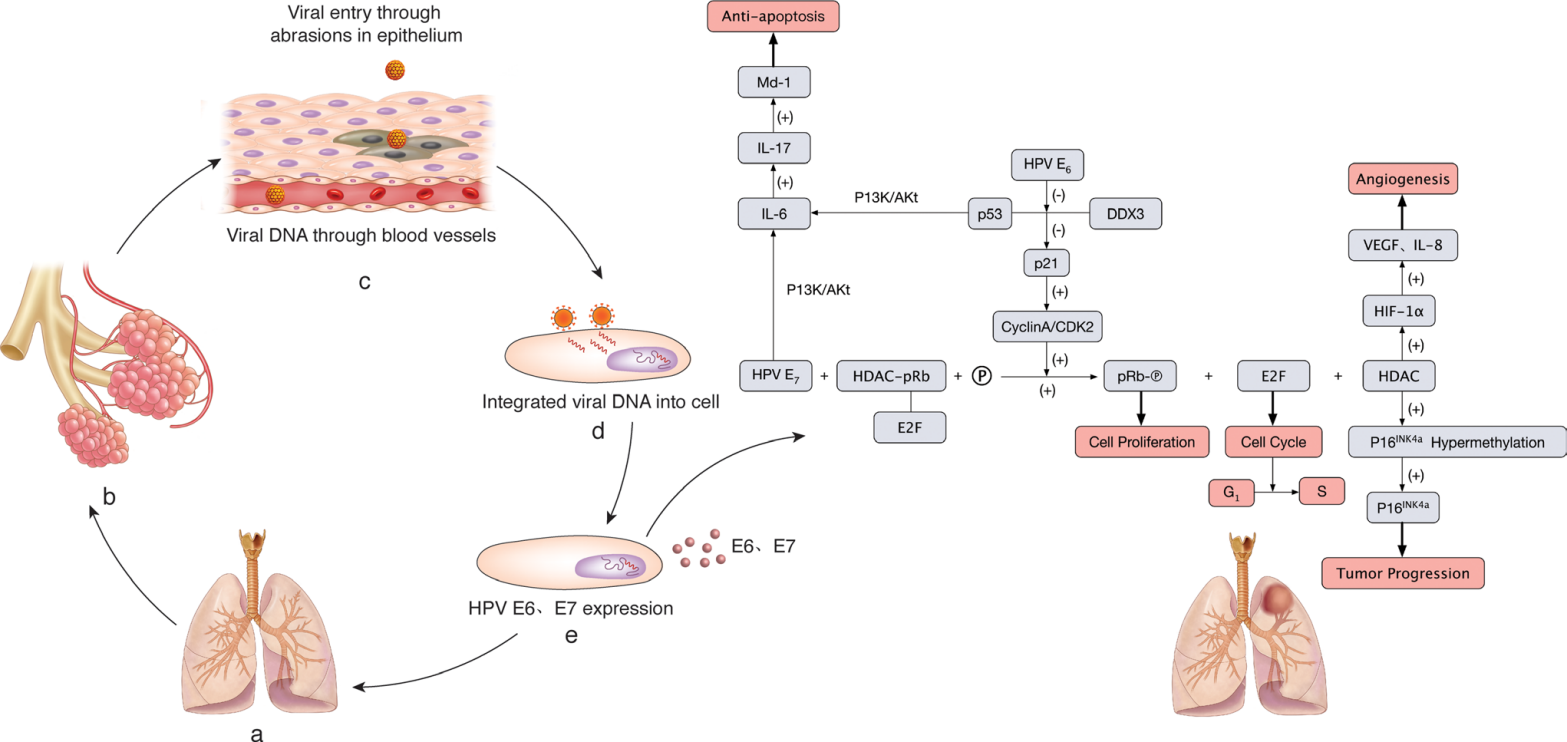
The molecular mechanism of HPV infection leading to lung cancer (**A**–**B**) Normal structure of lung and alveoli. (**C**) HPV DNA enters the lung epithelial cells through blood vessels and pulmonary lumina. (**D**) HPVs are recognized by membrane receptors, and viral DNA is integrated into the host cells. (**E**) The expression of HPV E6 and E7 oncogene proteins plays an important role in carcinogenesis. HPV E6 inhibits p53 interaction with DDX3, following p21 inactivation. Thus, the complex cyclin A/CDK2 is free to phosphorylate pRb, which promotes cell proliferation, and the E2F transcription factor is released and determines the cell cycle and G1/S transition. E7 HPV resolves the HDAC/pRb/E2F complex by interacting with pRb. Hence, HDAC is released to hypermethylate p16INK4 and inhibits the expression of p16INK4, which leads to tumor progression. HDAC can also cause angiogenesis induced by VEGF and IL-8 through HIF-1α. HPV E7 and inactivated p53 by HPV E6 can up-regulate Mcl-1 through the PI3K/Akt-(IL-6)-(IL-17) pathway, resulting in anti-apoptosis.

The molecular mechanism of HPV infection leading to lung cancer has in recent years been an active research field, and a number of reviews have described the pathogenesis in detail [52–56]. HPV E6 and E7 oncogene proteins can regulate the expression of multiple target genes and proteins such as p53, pRb, HIF-1α, VEGF, IL-6, IL-10, Mcl-1, Bcl-2, cIAP-2, EGFR, FHIT, hTERT, HER-2, ALK, ROS1 and AhR to promote lung cell proliferation, angiogenesis and cell immortalization through various signaling pathways [56–60] (Figure 5). Therefore, in HPV-associated lung cancers, these target genes and proteins may be potential therapeutic targets.

Growing evidence supports the link between HPV infection and lung cancer, but the relationship is still debatable. To date, seven independent systematic reviews and meta-analyses [10, 61–66] have evaluated and incorporated existing research evidence that HPV may be a risk factor for lung cancer. However, the studies above do not include the results of longitudinal studies (such as nested case-control and cohort studies) with high causal inference, and it is not clear that HPV infection had a causal link with lung cancer. In this paper, we present the prospective nested case-control studies and cohort studies of HPV infection and lung cancer for the first time to provide meta-analysis. The result suggests that HPV infection increase the risk of lung cancer, pooled *OR* and 95% *CI*: 3.64 (2.60–5.08). Zhai et al. [66] analyze the case-control studies of HPV infection and lung cancer and find that the combined *OR* is 5.67 (95% *CI*: 3.09–10.40, *P* < 0.001). Moreover, an international pooled analysis about HPV-associated lung cancers shows that pooled *OR* is 3.86 (95% *CI*: 2.87–5.19) [64]. However, allogeneic case-control studies and self-matched case-control studies are difficult to determine the time sequence. Individuals suffering from lung cancer with lower immunity are more susceptible to HPV, which may lead to false causal association. The pooled *OR* of this study is smaller, which is possibly due to the inclusion of nested case-control studies leading to weak correlations. The pooled *OR* of allogeneic case-control studies is significantly higher than that of nested case-control studies, and HPV infection do not increase the risk of primary lung cancer in nested case-control studies. The lack of association between HPV antibody positivity and increased risk of lung cancer can be explained as a high level of serum HPV antibodies reflecting a strong host immune response that prevents the recurrence or reactivation of HPV infection and thereby hinders the development of HPV-associated lung cancer [44]. This hypothesis should be explored and verified.

At present, the conclusion of HPV infection and lung cancer is still inconsistent. The possible influencing factors are: research population, test samples, sample processing methods and detection methods [42]. Therefore, the effect of HPV infection on lung cancer is discussed from the aspects of research area, gender, smoking status, detection method, HPV subtype, material, histological type, clinical stage and histological differentiation. The results of subgroup analyses suggest that research area, gender, smoking status, HPV subtype, histological type, clinical stage and histological differentiation may be the sources of total heterogeneity. The prevalence of HPV infection in lung cancer patients is quite different in published studies which may be due to geographical differences. A meta-analysis shows that the average HPV infection rate of lung cancer in the world is 26.5%, the lowest in North America (12.5%), the highest in China (including Taiwan) (37.7%), followed by other Asian regions (17.2%), and the result of meta regression suggests that there may be a high incidence area of HPV infection in lung cancer (*P* = 0.02) [62]. According to our subgroup analysis of the continent, the pooled *OR* of Asian is higher than European and American. It is closely related to the higher positive rate of HPV in lung cancer tissues in Asia (28.1[63]-35.7%[61]) than in other continents. HPV is a well-known carcinogen in a particularly virulent form of head and neck cancer in never smokers. We find that HPV-infected non-smokers (*OR* = 6.51, 95% *CI*: 3.78–11.21) have a higher risk of lung cancer than smokers. A meta-analysis of four case-control studies shows pooled *OR* in never-smokers is 4.78 (95% *CI*: 2.25–10.15) [65], which is similar to our results. Reported prevalence of HPV infection among non-smoking lung cancer patients in Asia [67] and Europe [63] is significantly higher than that among smoking lung cancer patients, but some studies find that there is no statistically significant difference of HPV infection rate in smokers and non-smokers [46, 63]. Whether HPV infection has synergistic effect with smoking on carcinogenesis remains controversial. The combined *OR*s of tissue detection methods (PCR, ISH, SB/DB and sequencing) are generally higher than those of serological detection methods (LBMA and BMSM), possibly due to the low amount of HPV in the blood circulation and the low sensitivity and specificity of serological detection methods. The sensitivity and false positive rate of PCR are higher than other methods [64, 68]. However, we do not find the pooled *OR* of PCR is higher than other methods, resulting from the selection of primers and the small sample sizes of other detection methods. In this study, HPV 16, HPV 18 and HPV 11 infection significantly increase the risk of lung cancer, while HPV 6 and HPV 31 infection are not significantly associated with lung cancer. A meta-analysis suggests that the average infection rates of HPV 16 and HPV 18 in lung cancer patients are 19.80% and 18.59%, compared with non-cancer controls, the combined *OR*s are 5.84 (95% *CI*: 3.14–10.86) and 4.29 (95% *CI*: 2.34–7.86) [66]. There is no significant difference of carcinogenic risk between HPV 16 and HPV 18 in lung cancer [66]. It is generally believed that low-risk types of HPV lead to benign lesions without the potential for malignance [69]. However, the role of HPV 11 and HPV 31 in lung cancer remains unclear, and the association between HPV subtype and lung cancer is worthy of further study. HPV replicates when infected keratinocytes are differentiated and does not release virus particles into the blood [70]. Therefore, we find that the combined *OR* of tissue is significantly higher than that of blood. The point estimated *OR* of lung squamous cell carcinoma (5.66) is slightly higher than that of lung adenocarcinoma (5.39). Because of the affinity to squamous cell, HPV infection rate of squamous cell carcinoma is higher than that of adenocarcinoma. The result is consistent with the results of Syrjänen [62] and Zhai [66]. In addition to traditional squamous cell carcinoma, we also find that HPV infection may be associated with adenocarcinoma and small cell carcinoma. Limited to the number of studies, the association with adenosquamous carcinoma and large cell carcinoma has not been found. The association between HPV and other histopathological types of lung cancer needs further study.

In this article, we ensure the high recall ratio through multi-databases and multi- approaches, and improve the precision ratio with the strict inclusion and exclusion criteria. Eligible studies are selected by two people who paid attention to control the quality as much as possible to reduce the search bias. Subgroup and sensitivity analyses show that the result of meta-analysis is stable and the conclusion is reliable. However, in the process of meta-analysis, there are still some limitations: (1) Potential bias cannot be completely ruled out, because HPV infection depends largely on the sensitivity, specificity and HPV subtype of detection methods. Subgroup analysis can explain partial bias, but there are still unknown bias. (2) There is a slight publication bias. According to the funnel plot, there is still a lack of small samples and no statistically significance unpublished articles. (3) There may be multiple publication bias, because it is difficult to distinguish whether a study published repeatedly.

In summary, our meta-analysis indicates that HPV infection, especially HPV 16 and 18, increases lung cancer risk, particularly in squamous cell carcinoma and small cell carcinoma. The development of international standard laboratory contributes to the favorable combination of multi-center experimental results and increases the reliability of causal inference. Case-control studies are difficult to determine the time sequence, so well-designed cohort study or randomized controlled trial urgent need to clarify the relationship between HPV and lung cancer. Although HPV vaccines can theoretically prevent the development of lung cancer, future research needs to focus attention toward whether an HPV vaccine can effectively reduce the incidence of lung cancer.

## MATERIALS AND METHODS

### Search strategy

We identified eligible studies either in English or Chinese published up to Feb 28, 2017 by searching the MEDLINE (PubMed), Embase (OVID) and Web of Science. Search terms were “human papillomavirus”, “HPV”, “lung carcinoma”, “lung neoplasm” and “lung lesions”. The search was limited to studies that had been conducted on human subjects. Meeting abstracts were excluded because of limited data they offered. Reference lists of the retrieved articles, reviews and editorials were also screened to find all additional eligible studies. This meta-analysis was performed in accordance with PRISMA guidelines.

### Study selection and inclusion criteria

The studies selected had to meet the following criteria: (1) case–control or cohort studies compared HPV infection among lung neoplasms patients and non-cancer controls; (2) histological diagnosis of cases and controls were established; (3) sufficient information was provided to calculate *OR* or *RR* with 95% *CI*; (4) there were no restrictions based on patients’ nationality, ethnicity or gender; (5) when an overlap of patients was found in several studies, only the study with the largest sample size and detailed information or the study that met the above criteria was included.

### Literature evaluation and data extraction

An initial screening of the title and abstract was performed in the first step, followed by a further screening based on a full text review. Information was independently extracted from all eligible publications by two investigators (Wei-min Xiong and Fei He), and discrepancies were resolved through discussion or via a third researcher. Literatures quality evaluation were evaluated using the Newcastle-Ottawascale (NOS) scale [71]. For studies meeting our inclusion criteria, the following data were collected: first author, publication year, country of study, specimen type, histological type, HPV detection method, HPV types, basic situation of case / exposure group and control / non-exposure group and numbers of HPV positive and negative subjects in tumors and control groups.

### Statistical analysis

When sufficient data were available, a meta-analysis was performed and *OR*s or *RR*s with corresponding 95% *CI*s were calculated. If there are no events in either the case or control arms of the trial, the trial should be discarded from the meta-analysis [72]. Heterogeneity among studies was examined using the Cochran's Q test by calculating the *P value* and *I*^2^ value[73]. If *I*^2^ is less than or equal to 50%, that suggested that it was homogeneity and the fixed effects model (Mantel-Haenszel method [74]) of meta-analysis was performed; if *I*^2^ > 50%, which means there is a statistical heterogeneity between the study results and the random effects model (DerSimonian-Laird method [75]) should be chosen [66].

Moreover, we did pre-specified exploratory meta-regression and subgroup analyses to investigate the effect of selected study and participant characteristics on the results, including study design, research area, gender, smoking status, detection methods, HPV subtypes, material, histological type, clinical stage and tissue differentiation. Sensitivity analysis by sequential omission of individual studies and trim and fill method [76] with recalculating of pooled *OR*/*RR*s and 95% *CI*s was conducted to test the meta-analysis results of stability. Using Contour-enhanced funnel plots (confunnel with filled studies from metatrim) [77], Beggs’ rank correlation test [78], Harbord's modified linear regression test [79] and Peters’ regression test [80] to detect funnel plot asymmetry and analyze potential publication bias. Rosenthal fail-safe number (Nfs) was also calculated to estimate the degree of publication bias and the meta-analysis results of stability [81]. Nfs_0.05_ = (Σz/1.64)^2^ - K, where z is the z value of each independent study and K is the number of studies. When Nfs > 5K + 10, it is judged that there is no publication bias [82].

All statistical tests were performed with the Stata 13.0 (Stata Corporation, College Station, TX, USA). All above analyses were two-sided test and the significance level was 0.05.

## SUPPLEMENTARY MATERIALS FIGURES AND TABLES



## Abbreviations

HPV: human papillomavirus
CI: confidence intervals
PCR: polymerase chain reaction
ISH: *in situ* hybridization
SB/DB: southern blot/dot blot
BMSM: bead-based multiplex serology method
LBMA: multiplex liquid bead microarray antibody assay
Nfs: Rosenthal fail-safe number
