# Supplementary material for: The association between human papillomavirus infection and lung cancer: a system review and meta-analysis
Source: Oncotarget. 2017 Oct 9;8(56):96419–32. doi: 10.18632/oncotarget.21682 (PMC5707111; doi:10.18632/oncotarget.21682)
Supplement: Supplementary file 1 [file oncotarget-08-96419-s001.pdf]

The association between human papillomavirus infection and lung cancer: a system review and meta-analysis

SUPPLEMENTARY MATERIALS

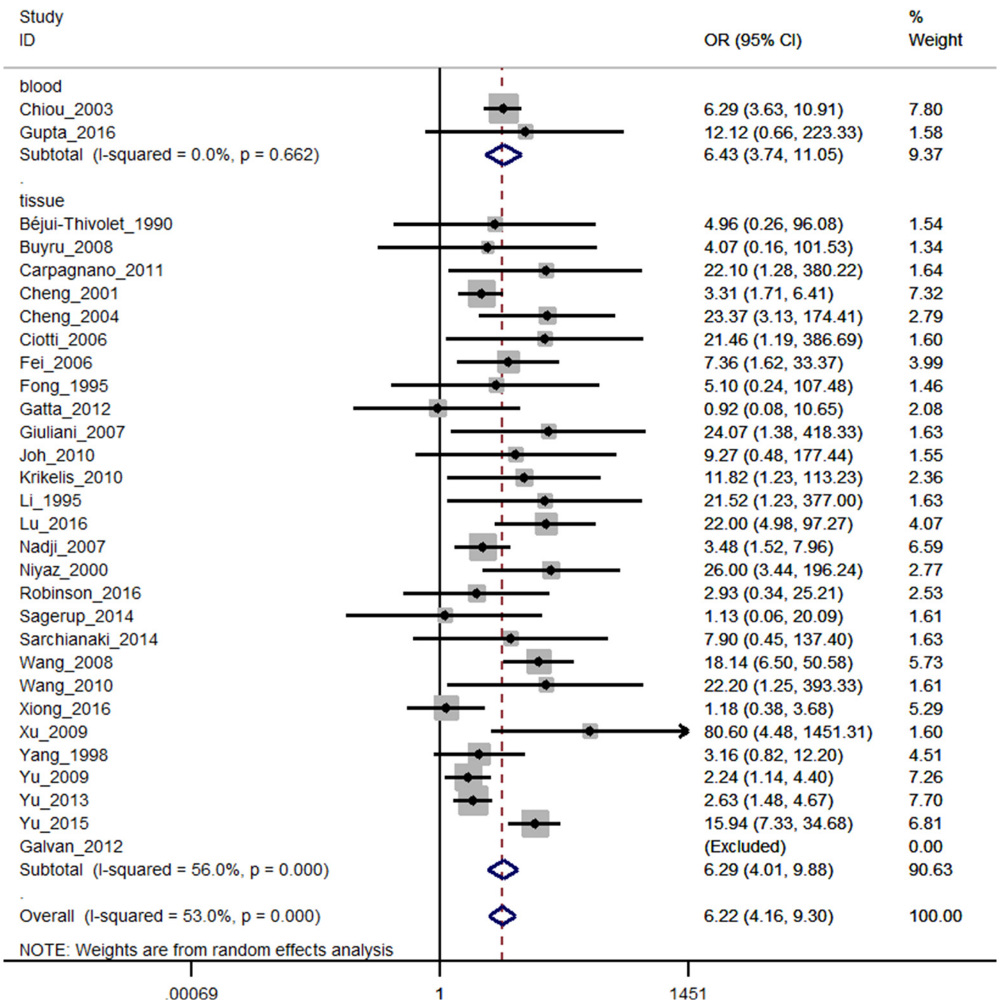

Supplementary Figure 1: Forest plot of HPV DNA positive rates in lung cancer patients and controls by sample type.
